# Large-Scale Modeling of Sparse Protein Kinase Activity Data

**DOI:** 10.1021/acs.jcim.3c00132

**Published:** 2023-06-09

**Authors:** Sohvi Luukkonen, Erik Meijer, Giovanni A. Tricarico, Johan Hofmans, Pieter F. W. Stouten, Gerard J. P. van Westen, Eelke B. Lenselink

**Affiliations:** † Leiden Academic Centre of Drug Research, Leiden University, Einsteinweg 55, 2333 CC Leiden, The Netherlands; ‡ Galapagos NV, Generaal De Wittelaan L11 A3, 2800 Mechelen, Belgium; ¶ Stouten Pharma Consultancy BV, Kempenarestraat 47, 2860 Sint-Katelijne-Waver, Belgium

## Abstract

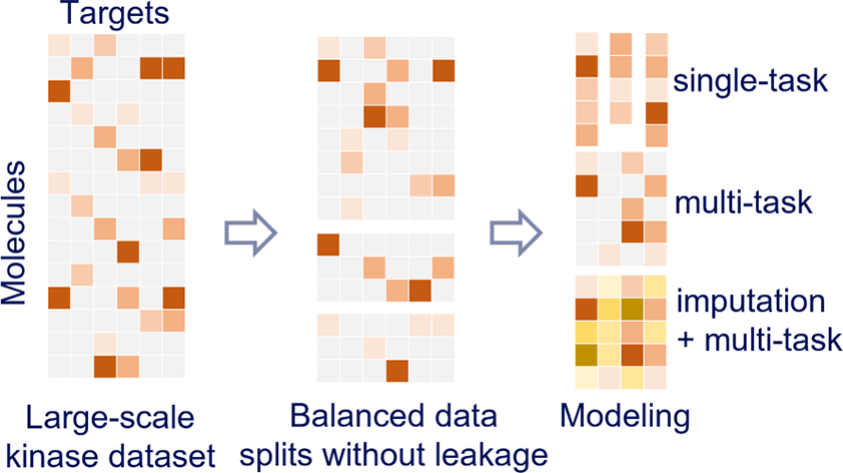

Protein kinases are a protein family that plays an important role in several complex diseases such as cancer and cardiovascular and immunological diseases. Protein kinases have conserved ATP binding sites, which when targeted can lead to similar activities of inhibitors against different kinases. This can be exploited to create multitarget drugs. On the other hand, selectivity (lack of similar activities) is desirable in order to avoid toxicity issues. There is a vast amount of protein kinase activity data in the public domain, which can be used in many different ways. Multitask machine learning models are expected to excel for these kinds of data sets because they can learn from implicit correlations between tasks (in this case activities against a variety of kinases). However, multitask modeling of sparse data poses two major challenges: (i) creating a balanced train–test split without data leakage and (ii) handling missing data. In this work, we construct a protein kinase benchmark set composed of two balanced splits without data leakage, using random and dissimilarity-driven cluster-based mechanisms, respectively. This data set can be used for benchmarking and developing protein kinase activity prediction models. Overall, the performance on the dissimilarity-driven cluster-based split is lower than on random split-based sets for all models, indicating poor generalizability of models. Nevertheless, we show that multitask deep learning models, on this very sparse data set, outperform single-task deep learning and tree-based models. Finally, we demonstrate that data imputation does not improve the performance of (multitask) models on this benchmark set.

## 1 Introduction

Protein kinases are a family of over 500 different enzymes responsible for protein phosphorylation. Most signaling pathways contain kinases, making them pivotal players in all aspects of protein regulation.^1,2^ They are found in animals, plants, bacteria, and archaea, indicating their importance to sustaining life, and their deregulation often leads to undesirable effects and pathologies.^3,4^ These include multiple forms of cancer and inflammatory, cardiovascular, immunological and infectious diseases.^5,6^ Thus, if the functioning of specific kinases in the body can selectively be altered, a wide range of diseases could potentially be treated, making protein kinases very interesting targets for drug discovery.

In the two decades since the FDA-approved imatinib more than 70 protein kinase inhibitors, mostly for applications in oncology, have been approved, and many more inhibitors are in (pre)clinical pipelines.^6,7^ Despite the success, there is still a need for better inhibitors that can selectively target either a single protein kinase, a subset of targets (so-called polypharmacological compounds, which can modulate multiple targets), or mutant protein kinases to address resistance. However, the development of a new drug from early stage drug discovery to clinical development is a challenging and expensive process that takes on average more than ten years and costs more than two billion dollars.^8^

Computer-aided drug design (CADD) can reduce these costs by decreasing the number of compounds to be synthesized and the number of experiments needed, especially when applied in early stage drug discovery. Moreover, CADD can enable early discontinuation of compounds predicted to fail. Machine learning-based quantitative structure–activity relationship (QSAR) models trained on experimental data are often used to predict activities from a compound’s 2D or 3D structure.^9^ Historically, QSAR models were single-task (ST), i.e., a single model was developed for activity against a single target. However, activities against targets with a conserved ATP binding site, such as protein kinases, are often correlated.^10−12^ Single-task models cannot take advantage of such correlations, but multitask (MT) models should be able to utilize these implicit correlations making the training process more efficient and the model predictions for each kinase more robust in that they suffer less from individual experimental errors and are applicable to a larger region of chemical space.^13−16^

A hurdle to developing good multitask models for activity predictions is the sparsity of the experimental data. Compounds with experimental data against multiple or ideally all targets are rare, making the data density of the molecules–targets matrix very low. Data imputation has been proposed as a solution.^17−19^ Imputation is the process of using predicted values for missing data points in the data set used to train the machine learning models. The complexity of the imputation strategy to obtain the predictions ranges from the simple computation of the mean value of the known data points per task to the use of deep learning models.^20^ Subsequently, the imputed values are used together with the experimental activity data to train the models.

In drug design, we aim for generalizable models, i.e., as much as possible they should predict the properties of novel compounds well. Therefore, model performance should be evaluated with a “realistic” split (i.e., to the maximum extent possible corresponding to real-life situations), where the chemical similarity between the training and test sets is minimized. For single-task modeling, this is often straightforward, but for multitask modeling, the construction of realistic balanced training–validation–test splits without data leakage between tasks is not as straightforward.^21^

If the splits are done per target, this will lead to data leakage, i.e., the same compound can be in the training set for one task and in the validation or test set for another one. As a consequence, training, validation, and test sets are overlapping. This in turn leads to an overestimation of the performance of the models. On the other hand, when a “general” random, cluster, or temporal split is applied to the overall data set, the data sets will be unbalanced (different ratios of compounds in training, validation, and test sets for the various tasks). Moreover, a random split does not result in chemical dissimilarity between the training and test sets, and as a consequence, a model’s generalizability and performance will be overestimated.^22^ A basic cluster split, where the clusters are combined to create the training–validation–test set without enforcing molecular dissimilarity, is a variant of the random split and works approximately equally well as a random split, whereas the temporal split on public data can often lead to extremely unbalanced splits, where some tasks have little to no data in a given set. Two out of the three challenges (balance and no data leakage) to create a good training–validation–test split for multitask modeling can be addressed with a random global equilibrated selection (RGES) which is introduced in this paper. All three of them (no data-leakage, balance, and dissimilarity) can be addressed with a dissimilarity-driven global balanced clustering (DGBC) split recently proposed by Tricarico et al.^21^ It simultaneously maximizes dissimilarity and balances the individual sets globally. Furthermore, an ensemble of best-performing models had a predictive accuracy exceeding that of single-dose kinase activity assays.^23^ Beyond classic single-task and multitask modeling, the cross-kinome correlations have been utilized in proteochemometric modeling,^24−26^ and multitask imputation models profile QSAR^27^ and Alchemite^20^ have shown promising results.

In this paper, we introduce two large, curated data sets of kinase activity values from the public domain:

- Kinase200 contains kinases 197 with at least 200 activity data points per kinase
- Kinase1000 contains kinases 74 with at least 1,000 activity data points per kinase

**Figure 1:**
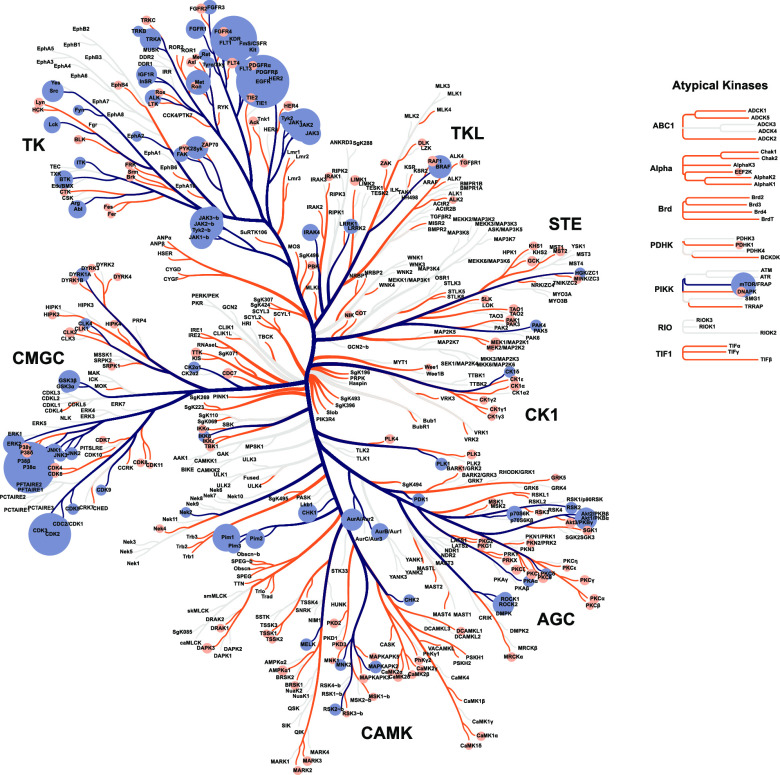
Human protein kinase tree where selected kinases are highlighted. Kinases in kinase1000 are in blue, and the extra kinases in kinase200 are in orange. The node size illustrates the number of compounds per selected kinase.

Selected kinases are highlighted on the human protein kinase tree in Figure 1.^28^ We also propose two well-balanced 80–10–10 multitask splits for activity prediction models: one based on random allocation and the other one on clustering.^21^ Finally, we benchmarked the capacity of different approaches to utilize large-scale protein kinase activity data to build prediction models. This was done by comparing the performance of a set of single-task models and multitask models with and without data imputation. All data and code are shared publicly so that they can be used as a benchmark set for building predictive protein kinase activity models.

## 2 Methods

### 2.1 Data

#### 2.1.1 Data Set Creation

The data sets were created based on the Papyrus data set (version 05.6).^31^ The Papyrus data set is a curated data set containing multiple large publicly available data sets, such as ChEMBL, and several smaller data sets. The data in the database have been standardized and normalized. Initially, all protein kinase activity data points (*K*_i_, *K*_D_, IC_50_, EC_50_) of kinases labeled as “high” quality were retrieved. Compounds with a molecular weight larger than 1000 Da, and activity points composed of multiple measurements with a standard deviation larger than 1.0 log units were filtered from the data set.

Furthermore, allosteric data points were removed based on text mining of ChEMBL assay descriptions and abstracts from PubChem, PubMed, CrossRef, and Google Patents for keywords. Additionally, we removed all compounds that had a Tanimoto similarity, calculated from Morgan fingerprints (3, 2048), above 0.8 to at least one of the molecules annotated as allosteric in the previous step.

The final data sets were constructed by selecting kinases with 200 or more data points (Kinase200) and with 1000 or more data points (Kinase1000), respectively. Unless mentioned otherwise, all following analyses and figures are done for the Kinase200 data set and corresponding figures for the Kinase1000 can be found in the Supporting Information. An overview of the data sets is summarized in Table 1.

**Table 1:** Properties of Our Two Protein Kinase Data Sets and Four Recently Published Kinase Data Sets

data set	no. kinases	no. molecules	no. data points	density
Kinase200	198	82,982	216,858	1.3%
Kinase1000	66	70,574	137,962	3.0%
PKIS^29^	224^a^	367	82,208	100%
Sharma et al.^30^	8	76,000	258,000	42%
pQSAR^27^	159^a^	13,190	114,317	5%
Born et al.^26^	349	113,475	206,989	0.5%

a Data points are labeled by assay instead of target.

#### 2.1.2 Data Splitting

The data sets were split into training, test, and validation sets using either random global equilibrated selection (RGES) split or dissimilarity-driven balanced cluster (DGBC) split with 80% of data going into the training set, 10% into the test set, and 10% into the validation set.

##### 2.1.2.1 Random Global Equilibrated Selection

The RGES split was done by sorting targets from the target with the most data points to those with the least. Then, for each target, a random split was made. If a compound belonged to a different (training, validation, test) set for a different target, its final label was set to the label of that compound for the target lowest on the sorted list. This mechanism was chosen because reassigning labels for targets with larger numbers of compounds has smaller relative effects on the balance.

##### 2.1.2.2 Dissimilarity-Driven Balanced Cluster

The DGBC split was made by using a method developed by Tricarico et al.^21^ First the compounds in the data set were clustered using sphere exclusion clustering on ECFP6 fingerprints with a Tanimoto distance of 0.736 between cluster centroids.^32^ Fingerprint generation and sphere exclusion clustering were done using RDKit (version 2020.09.05).^33^ The clusters were distributed over the training, validation, and test sets using linear programming to simultaneously achieve maximum dissimilarity between the sets and the desired training–validation–test ratio for each target.

##### 2.1.2.3 Evaluation of the Splits

Evaluation was done in three different ways:

- Data balance–data percentage per set and target
- Data distribution–distribution of pChEMBL values in each set
- Chemical dissimilarity–distribution of minimum Tanimoto distance of compound in each set compared to all compounds in the other sets

### 2.2 Models

The main aim of this work is twofold: (i) Assess to what extent accurate QSAR models can be developed on the basis of a large but sparse kinase-compound activity matrix. (ii) Assess to what extent imputation can improve the models. For this, we first developed four QSAR models and subsequently investigated whether data imputation can improve performance.

To develop baseline models, we used two well-known and widely used tree-based single-task methods: random forest and gradient boosting. The third model was a multitask version of gradient boosting. The fourth and fifth models were developed with directed message-passing neural networks (D-MPNN), as implemented in chemprop (CP).^34^ The D-MPNN approach was applied both to single-task and multitask models, referred to as CP_ST_ and CP_MT_, respectively. The sixth and seventh models applied the multitask D-MPNN approach where the missing values were imputed either by mean imputation or a single-task RF prediction, referred to as CP_MT_^Mean^ and CP_MT_^RF^, respectively. The eighth, and last, model was a reimplementation of the profile QSAR model (pQSAR) by Martin et al.^27^ The models were developed on a server containing 24 Intel(R) Xeon(R) CPU E5-2650 v4 @ 2.20 GHz CPUs, 7 NVIDIA GeForce GTX 1080 GPUs, and 1 NVIDIA GeForce RTX 2080 Ti GPU.

#### 2.2.1 Random Forest, XGBoost, and PyBoost

Sets of single-task RF_ST_ and XGB_ST_ models were developed for each kinase with the sklearn^35^ and xgboost^36^ packages, respectively, the multitask PB_MT_ model masking missing data in the loss function was developed with the pyboost^37^ packages. All three models used Morgan fingerprints as features (radius 3, 2048 bits). Initially, all models were developed with default parameters (RF: n_estimators=100, max_depth=None, min_sample_split=2, min_sample_leaf=1, max_features=1.0, XGB: ntrees=100, learning_rate=0.3, n_estimators=100, min_child_weight=1, colsample_bytree=1, scale_pos_weight=1, max_depth=6, subsample=1, PB: lr=0.05, min_gain_to_split=0, lambda_l2=1, gd_steps=1, max_depth=6, colsample=1, subsample=1, quantization=’Quantile’), and subsequently, hyperparameters of both sets of models were optimized on the validation set using a random grid search and selecting the best model based on the *R*^2^-score.

#### 2.2.2 Chemprop

Both a set of single-task (CP_ST_) models and a single multitask (CP_MT_) model were developed masking missing data in the loss function.^34^ The chemprop models were run both with default hyperparameters (hidden_size=300, depth=3, dropout=0.0, ffn_num_layers=300, activation=ReLU, bias=False, max_lr=1e-3, epochs=30) and with hyperparameters that were optimized on the validation set using Optuna.^38^ The optimized parameters were obtained from 150 trials of the data on five randomly chosen targets from the Kinase1000 data set split with BC split: P00533, P04626, P06239, Q5S007 and O75116.

#### 2.2.3 Chemprop with Data Imputation

Two chemprop multitask models with data imputation were also developed: one with mean imputation (CP_MT_^Mean^) and the other one with RF imputation (CP_MT_^RF^). In the former case, missing values are filled in by the arithmetic mean of the mean of available activities per compound and the mean of available activities per kinase, in the latter case, by making a single-task RF prediction. These new data sets with imputed values are then used to train a chemprop multitask model. Both models were run with default and optimized parameters.

#### 2.2.4 pQSAR

We reimplemented the pQSAR 2.0 method from Martin et al.^27^ in Python. In the first step missing data is imputed with single-task RF models. In the second step, for each kinase, a partial least-squares (PLS) regression model was developed with the experimental and imputed activity values of the other kinases as input data. As described in the pQSAR 2.0 paper, the number of components for PLS is selected based on an adjusted score by penalizing the *R*^2^ by 0.002 × number of components. Both the RF and PLS models were built with scikit-learn. The implementation was validated by training it on the data set accompanying the pQSAR paper by Martin et al. and comparing the results of our implementation to the results published in the paper. These results are shown in section S5.

In the original pQSAR paper, the train–test set split was done per assay instead of per kinase, so for some kinases, multiple different assays were included. In their implementation, the data split was done per task instead of on the complete data set. That led to data leakage between assays (Type-1 leakage), in that several compounds were in the training set for one assay and in the test set for another. Furthermore, during the PLS training process, the input data included both training and test compounds, leading to further data leakage (Type-2 leakage). As a consequence of this double leakage, the training and test sets overlapped, which in turn led to an overestimation of the performance of the models. In all work described here, we do not allow Type-1 data leakage. In order to assess the effect of Type-2 data leakage, we used our pQSAR implementation with the RGES and DGBC splits, respectively, both with and without including the test set during PLS training and comparing the performance of both approaches.

#### 2.2.5 Metrics

Predicted activity values were compared to experimental activity values by calculating the coefficient of determination (*R*^2^) and root-mean-squared error (RMSE) per kinase. Medians, means, and standard deviations of these distributions are reported.

## 3 Results and Discussion

### 3.1 Data Splits

We constructed two sets consisting of 80% (training)/10% (validation)/10% (test) sets for multitask modeling of protein kinases. These sets are well-balanced for all targets, and there is no data leakage between targets. The first set was created with the RGES split and the second with Tricarico’s DGBC split.

#### 3.1.1 Balanced Sets without Data Leakage

**Figure 2:**
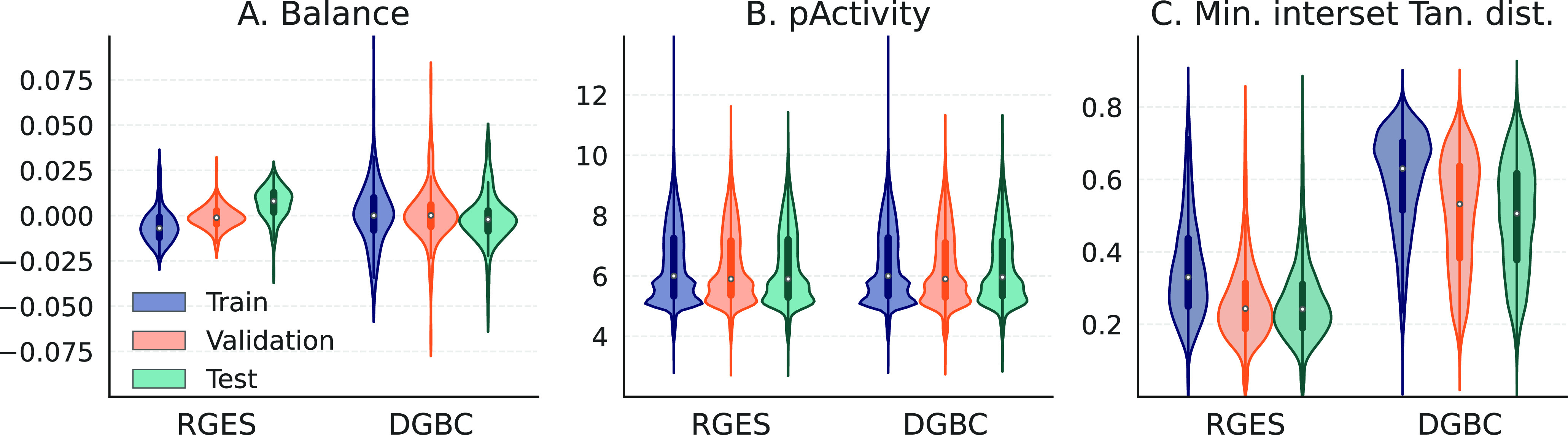
Set characteristics for RGES and DGBC splits. Distribution of (A) difference of data fraction to goal value per kinase, (B) pActivity values, and (C) minimum Tanimoto distance of molecules in a given set to molecules in the other two sets.

As illustrated in Figure 2A and summarized in Table S1, both data sets are well-balanced. For both splits, the mean of the ratio of the molecules per target is close to 80%/10%/10% and the standard deviations are small. The RGES split has a slightly more balanced ratio of molecules than the DGBC split. In Figure 2B, we show the pActivity (−log(activity)) value distribution per set. The distributions are very similar to each other indicating that activity values are also well-distributed between all sets.

#### 3.1.2 Sets for Interpolation and Extrapolation

For both splits, the chemical similarity of the sets is illustrated in Figure 2C, showing the distribution of the minimum Tanimoto distance of molecules (min(*d*_T_)) in a set to all the molecules in the other sets. The mean values per set are summarized in Table S1. As expected by design, the DGBC split yields more chemically dissimilar sets than the RGES split. This makes DGBC a more challenging split and therefore better suited for testing the generalizability of a model.

### 3.2 Modeling Kinase Activity

**Figure 3:**
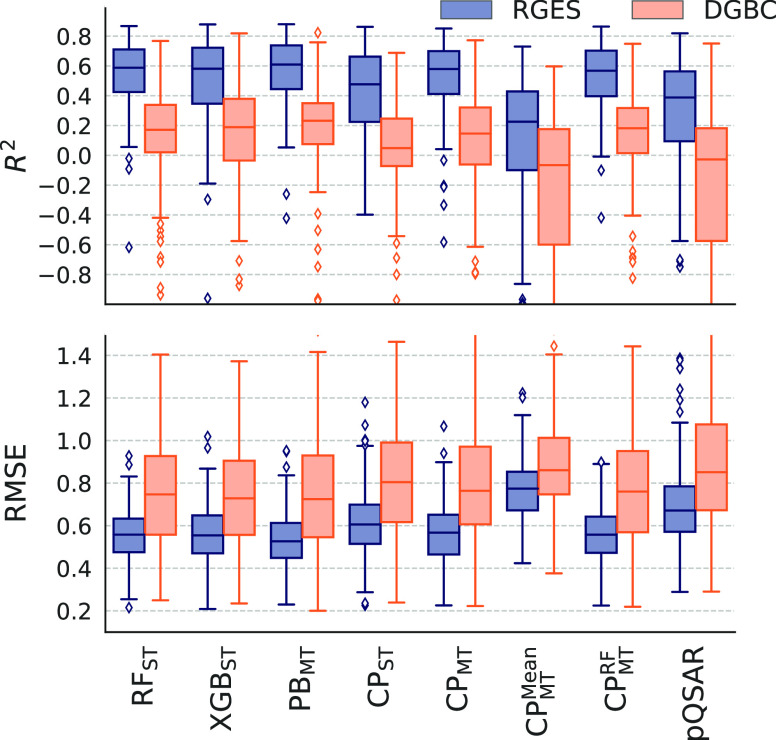
Comparison of the performance of the different models evaluated both with the random and structure-based splits. Distributions of *R*^2^ and RMSE values between predictions and experimental values of the data in the test set for each target kinase in the kinase200 data set split using either random global equilibrated selection (RGES, blue) and the dissimilarity-driven global balanced cluster (DGBC, orange) splits. Predictions were made using single-task random forest models (RF_ST_), xgboost (XGB_ST_) and chemprop (CP_ST_) models, multitask pyboost (PB), and chemprop multitask model without imputation (CP_MT_), with mean imputation (CP_MT_^Mean^), and with random forest imputation (CP_MT_^RF^), and profile QSAR (pQSAR).

We benchmarked the performance of a variety of well-known single-task and multitask ML models to model large-scale protein kinase activity data. All models have been evaluated with both the RGES and the DGBC splits. The overall results are summarized in Table 2 and Figure 3.

**Table 2:** Performance of Kinase Activity Prediction Models^a^

	*R*^2^				RMSE			
	median		mean (std)		median		mean (std)	
	RGES	DGBC	RGES	DGBC	RGES	DGBC	RGES	DGBC
RF_ST_	0.59	0.17	0.54 (0.22)	0.15 (0.32)	0.56	0.75	0.55 (0.13)	0.77 (0.30)
XGB_ST_	0.58	0.19	0.51 (0.27)	0.12 (0.64)	0.55	0.73	0.56 (0.13)	0.76 (0.30)
PB_MT_	**0.61**	**0.23**	**0.57** (0.21)	**0.20** (0.28)	**0.53**	**0.72**	**0.53** (0.13)	**0.75** (0.29)
CP_ST_	0.48	0.05	0.42 (0.30)	0.03 (0.40)	0.61	0.80	0.61 (0.16)	0.82 (0.25)
CP_MT_	0.58	0.15	0.52 (0.24)	0.08 (0.40)	0.57	0.76	0.56 (0.14)	0.78 (0.25)
CP_MT_^Mean^	0.23	–0.07	0.02 (0.68)	–0.37 (0.99)	0.77	0.86	0.77 (0.13)	0.89 (0.23)
CP_MT_^RF^	0.57	0.18	0.54 (0.22)	0.14 (0.33)	0.56	0.76	0.56 (0.13)	0.78 (0.1)
pQSAR	0.39	–0.03	0.26 (0.49)	–0.46 (2.14)	0.67	0.85	0.69 (0.21)	0.94 (0.50)

a Median, mean and standard deviations of *R*^2^ and RMSE metrics per kinase for single-task random forest (RF_ST_), xgboost (XGB_ST_), and chemprop single-task (CP_ST_) models, multitask pyboost model (PB_MT_), chemprop multitask models without data imputation (CP_MT_) and with mean (CP_MT_^Mean^) and RF imputation (CP_MT_^RF^), and the profile QSAR model without data leakage (pQSAR).

#### 3.2.1 Importance of Data Splitting

For all models, the performance is significantly better on the random-based split than on the structure-base splits. Most models reach median *R*^2^ > 0.6 and RMSE < 0.6, often used thresholds to consider a model to be predictive, based on an RGES split. However, on average the median *R*^2^ is 0.3 lower and the median RMSE is 0.2 higher for the DGBC split than the random split showing that the models do not perform as well on this data split. These results are in line with expectations and previously published results^21,39^ and show the importance to assess model performance with a realistic split.

#### 3.2.2 Hyperparameter Optimization Improves Performance

We optimized the hyperparameters of the tree- and chemprop-based models. The hyperparameters of the tree-based models were optimized separately for each model. To limit computation time, we only optimized the CP_MT_ model’s hyperparameters on the DGBC split and then applied the optimized parameters to all chemprop models. We show the performance of the optimized models in Figure 3 and Table 2, and the performances of the default models are summarized in Table S2.

On the RGES split, the hyperparameter optimization significantly increases the performance of all models, except the RF_ST_ models for which the performance does not change. The median *R*^2^ and RMSE were improved by 0.12 and 0.07 on average, respectively, and the largest improvements are seen for the PB_MT_) model (Δ*R*^2^ = 0.30 and ΔRMSE = 0.19). In the case of the DGBC split, the effect of the hyperparameter optimization is much smaller with the median *R*^2^ and RMSE only improving by 0.07 and 0.03 on average, respectively. The largest improvements are seen for PB_MT_ model (Δ*R*^2^ = 0.16 and ΔRMSE = 0.07) and the second largest for the XGB_ST_ models, indicating that hyperparameter optimization is necessary for gradient boosting models. It is surprising that the effect is consistently larger in the RGES than the DGBC split for the DMPNN-based models as the hyperparameter optimization was done with a small subset with the balanced cluster split. Nevertheless, all models were improved by hyperparameter optimization, and future studies could also use algorithms, like FABOLAS,^40^ that can handle hyperparameter optimization on very large data sets to yield even greater gain in performance for the multitask DMPNN models. The subsequent analyses in this paper were done with the optimized models.

#### 3.2.3 Multitask Models Outperform a Collection of Single-Task Models

To evaluate the effect utilizing correlations between kinases in sparse data, we compared pairwise the performance of single-task and multitask (i) gradient boosting models (XGB_ST_ vs PB_MT_) and (ii) chemprop models (CP_ST_ vs CP_MT_). For both cases and both splits, the multitask models are superior to the set of single-task models with increased average *R*^2^-scores and decreased RMSEs (Table 3).

**Table 3:** Effect of Multitask Modeling^a^

	XGB_ST_/PB_MT_		CP_ST_/CP_MT_	
	RGES	DGBC	RGES	DGBC
Δ⟨*R*^2^⟩	0.06	0.08	0.11	0.05
Δ⟨RMSE⟩	–0.03	–0.01	–0.05	–0.04

a Difference between the mean *R*^2^ and RMSE per kinase of single-task and multitask models.

The effects of multitask models are larger for the deep learning model compared to the gradient-boosting one. The CP models are based on learned representations; therefore on small, single-task data sets, the model is most likely not able to extract generalizable embeddings. The multitask model uses a much larger data set from which it is able to extract more generalizable embeddings, leading to a significant improvement. On the *R*^2^-score, on which we see the larger gains, the improvements are larger for RGES and DGBC splits in the case of deep learning and gradient boosting models, respectively. This indicates that the exploitation of intertarget correlation can be useful when predicting the activities of compounds dissimilar to the ones in the training set. Furthermore, in the case of the deep learning models, there is a speed-up of a factor ∼30 in computation time between running a single 198 multitask model and running 198 separate single-task models.

Similarly, in a recent study, Moriwaki et al.^41^ using an in-house data set with random splits for binary kinase activity predictions showed promising results for multitask graph neural networks that outperform single-task models. Another option to exploit the intertarget correlation is proteochemometric modeling.^42^ However, both our single-task and multitask models seem to outperform proteochemometric models used by Born et al. (RMSEs above 0.75 in a random split-based evaluation)^26^ in their large-scale kinase modeling.

#### 3.2.4 Tree-Based Machine Learning Outperforms Deep Learning

In the case of both single-task models (RF_ST_, XGB_ST_ vs CP_ST_) and multitask models (PB_ST_ vs CP_MT_), we see that the classical tree-based machine learning methods outperform the deep learning model. Even though in general deep learning models have been shown to outperform classic machine learning approaches in activity prediction,^43^ these results are in line with in-house results and other publications^23,44,45^ that show that in some cases classic ML methods perform as well as deep learning models.

The superior performance of tree-based methods over deep learning models has been well described on small- to medium-sized tabular data sets; several reasons for this are that NNs are biased toward overly smooth solutions, uninformative features are more problematic for NNs, and NNs are typically not rotationally invariant, as discussed in ref (46). Moreover, the deep learning approach is based on learned representations, therefore on small, single-task data sets, the model is most likely not able to extract generalizable embeddings. This would explain why the difference between the single-task models is larger than that between the multitask ones. Furthermore, the hyperparameters of each tree-based model were optimized separately, whereas, due to computation time, deep learning models use parameters optimized once on a subset of the multitask model so they might not be optimal for each separate model.

#### 3.2.5 Performance Is Not Correlated with Data Density

**Figure 4:**
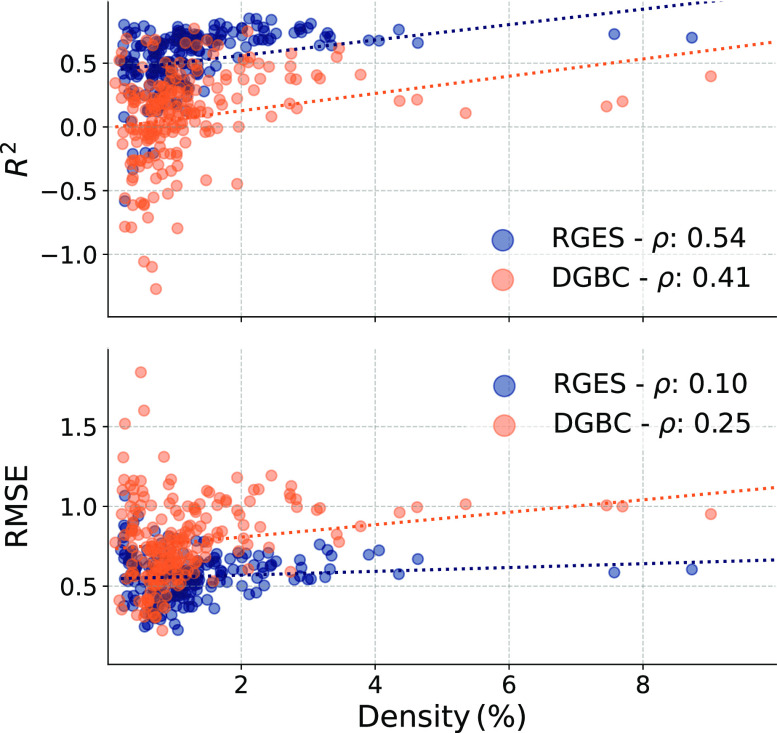
CP_MT_’s performance per kinase as a function of kinase’s data density. The coefficient of determination (*R*^2^) and root-mean-squared error (RMSE) between predictions and experimental values for the test set of each target kinase as a function of the data density of that target kinase. Results for random global equilibrated selection (RGES) split in blue and the dissimilarity-driven global balanced cluster (DGBC) split in orange.

We have evaluated whether there is a correlation between density and model performance and whether there is a difference in performance when using data sets at different density levels. In Figure 4, we illustrate that the performance of the CP_MT_ model for each kinase is poorly correlated with the data density points for that kinase, assessed by the *R*^2^ and the RMSE.

**Figure 5:**
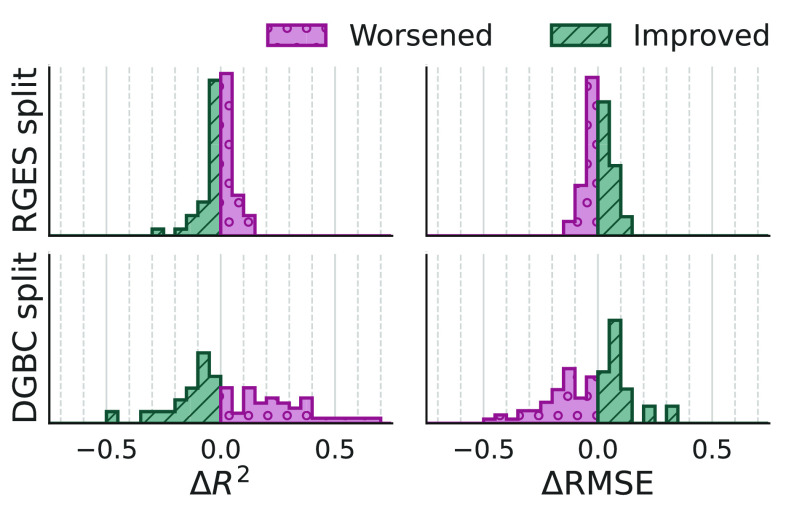
Difference of CP_MT_’s performance per kinase between the smaller and larger data set. Distributions of *R*^2^ = *R*_kinase1000_^2^ – *R*_kinase200_^2^ (top) and for RMSE = RMSE_kinase1000_ – RMSE_kinase200_ (bottom) for kinase present in both data sets. In green and purple, kinases for which model performance increases or decreases with the addition of sparse kinases to the data set.

To evaluate how the multitask model performance is affected by adding activity data on targets that have lower data density, the CP_MT_ was run on two data sets, kinase1000 and kinase200, which have different densities. The performance difference for targets that are present in both the kinase200 and kinase1000 data sets is shown by the distributions of Δ*R*^2^ = *R*_kinase1000_^2^ – *R*_kinase200_^2^ and ΔRMSE = RMSE_kinase1000_ – RMSE_kinase200_ in Figure 5. For the RGES split, it is clear that there is no difference in performance between the two data sets, so adding activity data from targets that have fewer data points does not improve the performance of the models on the other targets. For the DGBC split, there are larger differences in performance between sets, but there is no systematic improvement with the addition of the sparser kinases to the data set. Overall, adding more kinases with fewer data points leads to a sparser data matrix, and it does not improve model performance.

#### 3.2.6 Data Imputation Does Not Improve chemprop Performance

As the data set is very sparse, making it difficult to exploit interkinase correlation, we investigated if data imputation could improve the performance of the multitask models. Using chemprop, multitasking methods *with* imputation, CP_MT_^Mean^ and CP_MT_^RF^, does not show any improvement over multitasking *without* imputation CP_MT_ (Figure 3 and Table 2). CP_MT_^RF^ has very similar performance to CP_MT_, and CP_MT_^Mean^ underperforms significantly compared any other model, single-task or multitask.

#### 3.2.7 pQSAR without Data Leakage Underperforms

**Figure 6:**
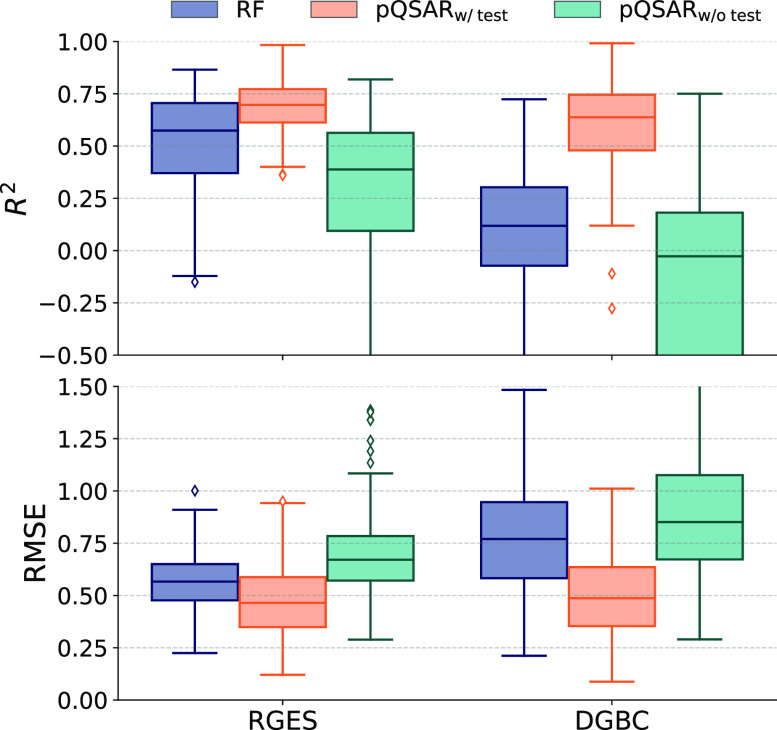
Performance of pQSAR performance with and without data leakage. Distributions of *R*^2^ and RMSE values between predictions and experimental values of the data in the test set for each target kinase in the kinase200 data set. Predictions were made using single-task random forest models (RF_ST_), pQSAR with PLS trained on training and test set (pQSAR_w/test_), and profile QSAR with PLS trained on the training set only (pQSAR_w/o test_). Results are shown for both the random global equilibrated split (RGES) and dissimilarity-driven global balanced cluster (DGBC) split.

In Martin et al.,^27^ the test set is included when training the PLS model which leads to data leakage between kinases. To investigate the impact of this, we trained the PLS both with and without the test set. In Figure 6 and Table S3, we show that when the test set is not included in the training of the PLS, the performance measures turn out worse than when the test set is included. The pQSAR without the test set performs worse than the single-task RF_ST_ sets, and when the test set is included in training it outperforms the RFs. The test set should be independent so we exclude the test set from the training of the PLS models. In this scenario, the pQSAR model underperforms compared to every other model except CP_MT_^Mean^. Alchemite, a commercial deep learning-based method using imputation adopts an expectation-maximization algorithm and has shown promising results on the cluster-based pQSAR data set as well, but as mentioned before the splits from pQSAR suffer from data leakage.^20^

## 4 Conclusion

In this study, we have investigated the large-scale modeling of protein kinase activity with various machine-learning approaches. For this, we created two large protein kinase data sets from the curated Papyrus database,^31^ comprising 198 kinases and 83K molecules (kinase200) and 66 kinases and 71K molecules (kinase1000), respectively. Other recently applied kinase data sets contain a more limited number of either targets^30^ or molecules,^27,29^ except for the data set based on all human kinases used by Born et al.^26^ For each of our data sets, two balanced multitask splits without data leakage between targets were created, a random (RGES) and a dissimilarity-driven cluster-based (DGBC) split, to evaluate rigorously the performance of the models within exploitation and exploration schemes. Other publications of large-scale kinase modeling either have been using only random-based splits^26^ or have had data leakage between targets.^27^

We then compared the performance of seven models to predict protein kinase activity. These included sets of single-task random forest, xgboost, and chemprop models, a multitask pyboost model, chemprop models without and with data imputation, and our implementation of pQSAR 2.0. In the cases of both single-task and multitask models, we see that the deep learning-based models are outperformed by classic machine learning methods on both splits. The performance of gradient boosting and D-MPNN models on both splits can be improved by creating multitask models, indicating that exploitation of intertarget correlation can be useful when predicting activities of compounds both similar and dissimilar to the ones in the training set. The multitask model’s performance could not be improved by data imputation. Moreover, using pQSAR without data leakage between targets also does not lead to higher predictive power.

As expected, we see that every model performs significantly worse with the dissimilarity-driven split than with the random split. Most of the models show some predictive power with the random split but struggle with the cluster-based split. The dissimilarity-driven cluster-based split is a more conservative estimation of performance, and thus it is a more realistic assessment of the performance of machine learning models in real drug discovery projects. As the poor performance on the cluster-based split of the different models shows (for the PB_MT_, the overall best-performing model, only 19% of kinase have an *R*^2^ above 0.4), further developments are needed to efficiently model activity with large-scale sparse data sets. The inclusion of additional data in the form of protein information may improve this performance.

## Data Availability

The code and the data sets are provided to the community at https://github.com/CDDLeiden/kinase-modelling as a benchmark set for developing large-scale multitask models for kinases.

## Supporting Information Available

S1, Extra analysis of the kinase200 split; S2, analysis of the kinase1000 splits; S3, models’ performance with default parameters on kinase200 set; S4, models’ performance with default parameters on kinase1000 set; S5, pQSAR implementation validation (PDF)

## Glossary

density: percentage of a data matrix filled with non-null elements (also referred to as data density)
CP: chemprop
DGBC: dissimilarity-driven global balance clustering
DMPNN: directed message passing neural network
MT: multitask
PB: pyboost
*R*^2^: coefficient of determination
RF: random forest
RGES: random global equilibrated selection
RMSE: root-mean-squared error
ST: single-task
XGB: xgboost
