# Supplementary material for: Large-Scale Modeling of Sparse Protein Kinase Activity Data
Source: J Chem Inf Model. 2023 Jun 9;63(12):3688–96. doi: 10.1021/acs.jcim.3c00132 (PMC10302492; doi:10.1021/acs.jcim.3c00132)
Supplement: Supplementary file 1 — ci3c00132_si_001.pdf [file ci3c00132_si_001.pdf]

Large-scale modelling of sparse protein kinase activity data

Sohvi Luukkonen,[†] Erik Meijer,[†] Giovanni A. Tricarico,[‡] Johan Hofmans,[‡] Pieter F.W. Stouten,[‡,†,¶] Gerard J.P. van Westen,[*,†] and Eelke B. Lenselink[*,‡]

†*Leiden Academic Centre of Drug Research, Leiden University, Einsteinweg 55, 2333 CC Leiden, The Netherlands*

‡*Galapagos NV, Generaal De Wittelaan L11 A3, 2800 Mechelen, Belgium*

¶*Stouten Pharma Consultancy BV, Kempenarestraat 47, 2860 Sint-Katelijne-Waver, Belgium*

E-mail: gerard@lacdr.leidenuniv.nl; bart.lenselink@glpg.com

Abstract

Protein kinases are a protein family that plays an important role in several complex diseases such as cancer, and cardiovascular and immunological diseases. Protein kinases have conserved ATP binding sites, which when targeted can lead to similar activities of inhibitors against different kinases. This can be exploited to create multi-target drugs. On the other hand, selectivity (lack of similar activities) is desirable in order to avoid toxicity issues. There is a vast amount of protein kinase activity data in the public domain, which can be used in many different ways. Multi-task machine learning models are expected to excel for these kinds of datasets because they can learn from implicit correlations between tasks (in this case activities against a variety of kinases). However, multi-task modelling of sparse data poses two major challenges: *(i)* creating a balanced train-test split without data leakage and *(ii)* handling missing data. In this work, we construct a protein kinase benchmark set composed of two balanced splits without data leakage, using random and dissimilarity-driven cluster-based mechanisms, respectively. This dataset can be used for benchmarking and developing protein kinase activity prediction models. Overall, the performance on the dissimilarity-driven cluster-based split is lower than on random split-based sets for all models, indicating poor generalizability of models. Nevertheless, we show that multi-task deep learning models, on this very sparse dataset, outperform single-task deep learning and tree-based models. Finally, we demonstrate that data imputation does not improve the performance of (multi-task) models on this benchmark set.

1 Introduction

Protein kinases are a family of over 500 different enzymes responsible for protein phosphorylation. Most signalling pathways contain kinases making them pivotal players in all aspects of protein regulation.[1,2] They are found in animals, plants, bacteria and archaea, indicating their importance to sustaining life and their deregulation often leads to undesirable effects and pathologies.[3,4] These include multiple forms of cancer, inflammatory, cardiovascular, immunological and infectious diseases.[5,6] Thus, if the functioning of specific kinases in the body can selectively be altered, a wide range of diseases could potentially be treated, making protein kinases very interesting targets for drug discovery.

In the two decades since the FDA-approved imatinib more than 70 protein kinase inhibitors, mostly for applications in oncology, have been approved and many more inhibitors are in (pre-)clinical pipelines.[6,7] Despite the success, there is still a need for better inhibitors that can either selectively target a single protein kinase, a subset of targets (so-called poly-pharmacological compounds, which can modulate multiple targets), or mutant protein kinases to address resistance. However, the development of a new drug from early-stage drug discovery to clinical development is a challenging and expensive process that takes on average more than ten years and costs more than two billion dollars.[8]

Computer-aided drug design (CADD) can reduce these costs by decreasing the number of compounds to be synthesised and the number of experiments needed, especially when applied in early-stage drug discovery. Moreover, CADD can enable early discontinuation of compounds predicted to fail. Machine learning-based quantitative structure-activity relationship (QSAR) models trained on experimental data are often used to predict activities from a compound's 2D or 3D structure.[9] Historically, QSAR models were single-task (ST), *i.e.* a single model was developed for activity against a single target. However, activities against targets with a conserved ATP binding site, such as protein kinases, are often correlated.[10–12] Single-task models cannot take advantage of such correlations, but multi-task (MT) models should be able to utilise these implicit correlations making the training process more efficient and the model predictions for each kinase more robust in that they suffer less from individual experimental errors and are applicable to a larger region of chemical space.[13–16]

A hurdle to developing good multi-task models for activity predictions is the sparsity of the experimental data. Compounds with experimental data against multiple or ideally all targets are rare, making the data density of the molecules-targets matrix very low. Data imputation has been proposed as a solution.[17–19] Imputation is the process of using predicted values for missing data points in the dataset used to train the machine learning models. The complexity of the imputation strategy to obtain the predictions ranges from the simple computation of the mean value of the known data points per task to the use of deep learning models.[20] Subsequently, the imputed values are used together with the experimental activity data to train the models.

In drug design we aim for generalizable models, *i.e.* as much as possible they should predict the properties of novel compounds well. Therefore, model performance should be evaluated with a 'realistic' split (*i.e.* to the maximum extent possible corresponding to real-life situations), where the chemical similarity between the training and test sets is minimised. For single-task modelling, this is often straightforward, but for multi-task modelling, the construction of realistic balanced training-validation-test splits without data leakage between tasks is not as straightforward.[21]

If the splits are done per target, this will lead to data leakage, *i.e.* the same compound can be in the train for one task and in the validation or test set for another one. As a consequence, training, validation and test sets are overlapping. This in turn leads to an overestimation of the performance of the models. On the other hand, when a 'general' random, cluster, or temporal split is applied to the overall dataset, the datasets will be unbalanced (different ratios of compounds in training, validation and test sets for the various tasks). Moreover, a random split does not result in chemical dissimilarity between the training and test sets, and as a consequence, a model's generalizability and performance will be overestimated.[22] A ba-

sic cluster split, where the clusters are combined to create the training-validation-test set without enforcing molecular dissimilarity, is a variant of the random split and works approximately equally well as a random split. Whereas the temporal split on public data can often lead to extremely unbalanced splits, where some tasks have little to no data in a given set. Two out of the three challenges (balance and no data leakage) to create a good training-validation-test split for multi-task modelling can be addressed with a random global equilibrated selection (RGES) which is introduced in this paper. All three of them (no data-leakage, balance and dissimilarity) can be addressed with a dissimilarity-driven global balanced clustering (DGBC) split recently proposed by Tricarico et al.[21] It simultaneously maximises dissimilarity and balances the individual sets globally. Furthermore, an ensemble of best-performing models had a predictive accuracy exceeding that of single-dose kinase activity assays.[23] Beyond, classic single- and multi-task modelling, the cross-kinome correlations have been utilised in proteochemometric modelling,[24–26] and multi-task imputation models profile QSAR[27] and Alchemite[20] have shown promising results.

In this paper, we introduce two large, curated datasets of kinase activity values from the public domain:

- Kinase200 contains kinases 197 with at least 200 activity data points per kinase
- Kinase1000 contains kinases 74 with at least 1,000 activity data points per kinase

Selected kinases are highlighted on the human protein kinase tree in Figure 1.[28] We also propose two well-balanced 80-10-10 multi-task splits for activity prediction models: one based on random allocation and the other one on clustering.[21] Finally, we benchmarked the capacity of different approaches to utilise large-scale protein kinase activity data to build prediction models. This was done by comparing the performance of a set of single-task models and multi-task models with and without data imputation. All data and code are shared publicly so that they can be used as a benchmark set for building predictive protein kinase activity models.

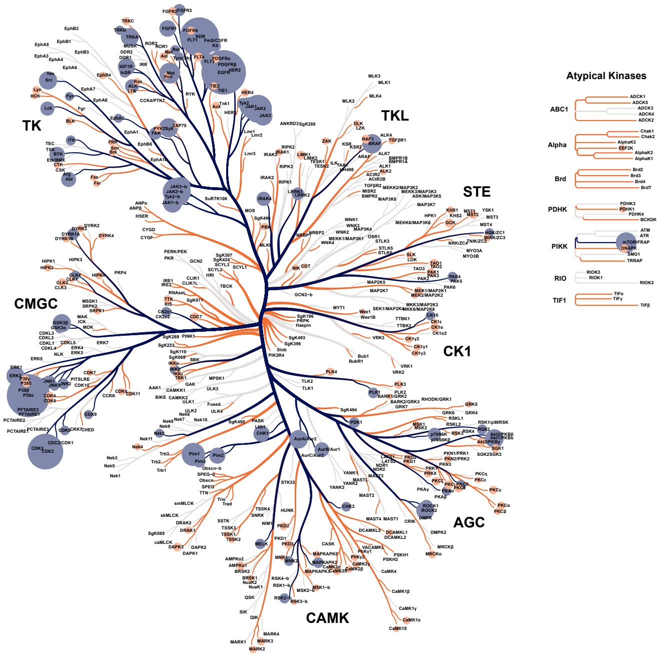

Figure 1: Human protein kinase tree where selected kinases are highlighted. Kinases in kinase1000 are in blue and the extra kinases in kinase200 are in orange. The node size illustrates the number of compounds per selected kinase.

2 Methods

2.1 Data

2.1.1 Dataset creation

The datasets were created based on the Papyrus dataset (version 05.6).[31] The Papyrus dataset is a curated dataset containing multiple large publicly available datasets, such as ChEMBL, and several smaller datasets. The data in the database have been standardised and normalised. Initially, all protein kinase activity data points (Ki, KD, IC50, EC50) of kinases labelled as 'high' quality were retrieved. Compounds with a molecular weight larger than 1000 Da, and activity points composed of multiple measurements with a standard deviation larger than 1.0 log units were filtered from the dataset.

Furthermore, allosteric data points were removed based on text mining of ChEMBL assay descriptions and abstracts from PubChem, PubMed, CrossRef and Google Patents for keywords. Additionally, we removed all compounds that had a Tanimoto similarity, calculated from Morgan fin-

Table 1: Properties of our two protein kinase datasets and four recently published kinase datasets. [a] Data points are labelled by assay instead of target.

Dataset	#kinases	#molecules	#data points	Density
Kinase200	198	82,982	216,858	1.3%
Kinase1000	66	70,574	137,962	3.0%
PKIS[29]	224[a]	367	82,208	100%
Sharma et al.[30]	8	76,000	258,000	42%
pQSAR[27]	159[a]	13,190	114,317	5%
Born et al.[26]	349	113,475	206,989	0.5%

gerprints (3, 2048), above 0.8 to at least one of the molecules annotated as allosteric in the previous step.

The final datasets were constructed by selecting kinases with 200 or more data points (Kinase200) and with 1000 or more data points (Kinase1000), respectively. Unless mentioned otherwise, all following analyses and figures are done for the Kinase200 dataset and corresponding figures for the Kinase1000 can be found in the Supplementary Information. An overview of the datasets is summarised in Table 1.

2.1.2 Data splitting

The datasets were split into training, test and validation sets using either random global equilibrated selection (RGES) split or dissimilarity-driven balanced cluster (DGBC) split with 80% of data going into the training set, 10% into the test set and 10% into the validation set.

Random global equilibrated selection (RGES) split was done by sorting targets from the target with the most data points to those with the least. Then, for each target, a random split was made. If a compound belonged to a different (training, validation, test) set for a different target, its final label was set to the label of that compound for the target lowest on the sorted list. This mechanism was chosen because reassigning labels for targets with larger numbers of compounds has smaller relative effects on the balance.

Dissimilarity-driven balanced cluster (DGBC) split was made by using a method developed by Tricarico et al.[21] First the compounds in the dataset were clustered using sphere exclusion clustering on ECFP6 fingerprints with a Tanimoto distance of 0.736 between cluster centroids.[32] Fingerprint generation and sphere exclusion clustering were done using RDKit (version 2020.09.05).[33] The clusters were distributed over the training, validation and test sets using linear programming to simultaneously achieve maximum dissimilarity between the sets and the desired training-validation-test ratio for each target.

Evaluation of the splits was done in three different ways:

- Data balance – data percentage per set and target
- Data distribution – distribution of pChEMBL values in each set
- Chemical dissimilarity – distribution of minimum Tanimoto distance of compound in each set compared to all compounds in the other sets

2.2 Models

The main aim of this work is two-fold: *(i)* Assess to what extent accurate QSAR models can be developed on the basis of a large but sparse kinase-compound activity matrix. *(ii)* To what extent imputation can improve the models? For this, we first developed four QSAR models and subsequently investigated whether data imputation can improve performance.

To develop baseline models, we used two well-known and widely used tree-based single-task methods: random forest and gradient boosting. The third model was a multi-task version of

gradient boosting- The fourth and fifth models were developed with directed message-passing neural networks (D-MPNN), as implemented in the chemprop (CP).[34] The D-MPNN approach was applied both to single- and multi-task models, referred to as $\mathrm{CP_{ST}}$ and $\mathrm{CP_{MT}}$, respectively. The sixth and seventh models applied the multi-task D-MPNN approach where the missing values were imputed either by mean imputation or a single-task RF prediction, referred to as $\mathrm{CP_{MT}^{Mean}}$ and $\mathrm{CP_{MT}^{RF}}$, respectively. The eighth, and last, model was a reimplementation of the profile QSAR model (pQSAR) by Martin et al.[27] The models were developed on a server containing 24 Intel(R) Xeon(R) CPU E5-2650 v4 @ 2.20GHz CPUs, 7 NVIDIA GeForce GTX 1080 GPUs and 1 NVIDIA GeForce RTX 2080 Ti GPU.

Random Forest, XGBoost and PyBoost Sets of single-task $\mathrm{RF_{ST}}$ and $\mathrm{XGB_{ST}}$ models were developed for each kinase with the sklearn[35] and xgboost[36] packages respectively, the multi-task $\mathrm{PB_{MT}}$ model masking missing data in the loss function was developed with the pyboost[37] packages. All three models used Morgan fingerprints as features (radius 3, 2048 bits). Initially, all models were developed with default parameters (RF: `n_estimators=100`, `max_depth=None`, `min_sample_split=2`, `min_sample_leaf=1`, `max_features=1.0`, XGB: `ntrees=100`, `learning_rate=0.3`, `n_estimators=100`, `min_child_weight=1`, `colsample_bytree=1`, `scale_pos_weight=1`, `max_depth=6`, `subsample=1`, PB: lr=0.05, min_gain_to_split=0, lambda_l2=1, gd_steps=1, max_depth=6, colsample=1, subsample=1, quantization='Quantile') and subsequently, hyperparameters of both sets of models were optimised on the validation set using a random grid search and selecting the best model based on the R^2-score.

Chemprop Both a set of single-task ($\mathrm{CP_{ST}}$) models and a single multi-task ($\mathrm{CP_{MT}}$) model were developed masking missing data in the loss function.[34] The chemprop models were run with both default hyperparameters (`hidden_size=300`, `depth=3`, `dropout=0.0`, `ffn_num_layers=300`, `activation=ReLU`, `bias=False`, `max_lr=1e-3`, `epochs=30`) and with hyperparameters that were optimised on the validation set using Optuna.[38] The optimised parameters were obtained from 150 trials of the data on five randomly chosen targets from the Kinase1000 dataset split with BC split: P00533, P04626, P06239, Q5S007 and O75116.

Chemprop with data imputation Two chemprop multi-task models with data imputation were also developed: one with mean imputation ($\mathrm{CP_{MT}^{Mean}}$) and the other one with RF imputation ($\mathrm{CP_{MT}^{RF}}$). In the former case, missing values are filled in by the arithmetic mean of the mean of available activities per compound and the mean of available activities per kinase. In the latter case, by making a single-task RF prediction. These new datasets with imputed values are then used to train a chemprop multi-task model. Both models were run with default and optimised parameters, respectively.

pQSAR We reimplemented the pQSAR 2.0 method from Martin, et al.[27] in Python. In the first step missing data is imputed with single-task RF models. In the second step, for each kinase, a partial least squares (PLS) regression model was developed with the experimental and imputed activity values of the other kinases as input data. As described in the pQSAR 2.0 paper, the number of components for PLS is selected based on an adjusted score by penalising the R^2 by 0.002 * number of components. Both the RF and PLS models were built with scikit-learn. The implementation was validated by training it on the dataset accompanying the pQSAR paper by Martin et al., and comparing the results of our implementation to the results published in the paper. These results are shown in section S5.

In the original pQSAR paper, the train-test set split was done per assay instead of per kinase, so for some kinases, multiple different assays were included. In their implementation, the data split was done per task instead of on the complete dataset. That led to data leakage between assays (Type-1 leakage), in that several compounds were in the training set for one assay and in the test set for another. Furthermore, during the PLS training

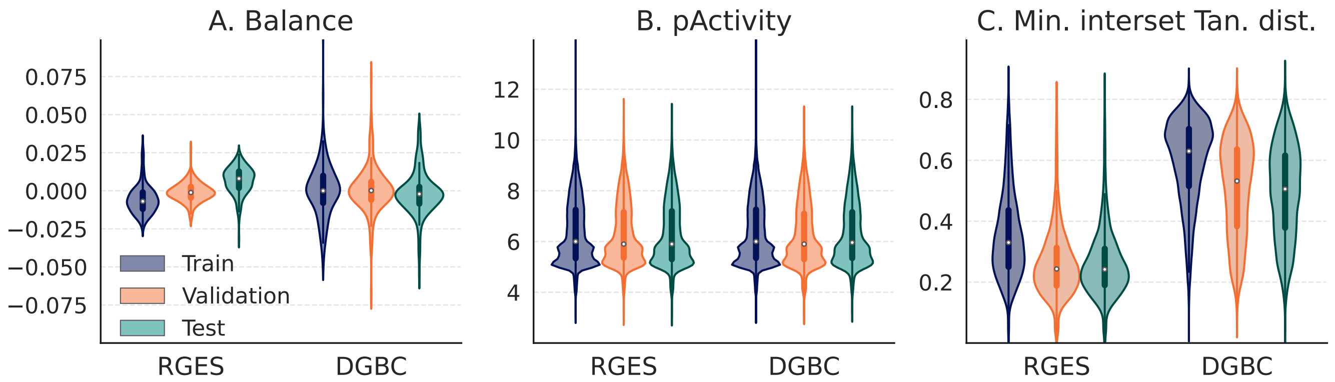

Figure 2: Set characteristics for RGES and DGBC splits. Distribution of **A.** difference of data fraction to goal value per kinase, **B.** pActivity values and **C.** minimum Tanimoto distance of molecules in a given set to molecules in the other two sets.

process, the input data included both training and test compounds, leading to further data leakage (Type-2 leakage). As a consequence of this double leakage, the training and test sets overlapped, which in turn led to an overestimation of the performance of the models. In all work described here, we do not allow Type-1 data leakage. In order to assess the effect of Type-2 data leakage, we used our pQSAR implementation with the RGES and DGBC splits, respectively, both with and without including the test set during PLS training and comparing the performance of both approaches.

Metrics Predicted activity values were compared to experimental activity values by calculating the coefficient of determination (R^2) and root-mean-squared error (RMSE) per kinase. Medians, means and standard deviations of these distributions are reported.

3 Results and discussion

3.1 Data splits

We constructed two sets consisting of 80% (training)/ 10% (validation)/ 10% (test) sets for multi-task modelling of protein kinases. These sets are well-balanced for all targets and there is no data leakage between targets. The first set was created with the RGES split and the second with Tricarico's DGBC split.

3.1.1 Balanced sets without data leakage

As illustrated in Figure 2A and summarised in Table S1, both datasets are well-balanced. For both splits, the mean of the ratio of the molecules per target is close to 80%/10%/10% and the standard deviations are small. The RGES split has a slightly more balanced ratio of molecules than the DGBC split. In Figure 2B, we show the pActivity (-log(activity)) value distribution per set. The distributions are very similar to each other indicating that activity values are also well-distributed between all sets.

3.1.2 Sets for interpolation and extrapolation

For both splits, the chemical similarity of the sets is illustrated in Figure 2C, showing the distribution of the minimum Tanimoto distance of molecules ($\min(d_T)$) in a set to all the molecules in the other sets. The mean values per set are summarised in Table S1. As expected by design, the DGBC split yields more chemically dissimilar sets than the RGES split. This makes DGBC a more challenging split, and therefore better suited for testing the generalizability of a model.

3.2 Modelling kinase activity

We benchmarked the performance of a variety of well-known single- and multi-task ML models to model large-scale protein kinase activity data. All models have been evaluated both with the RGES

and the DGBC splits. The overall results are summarised in Table 2 and Figure 3.

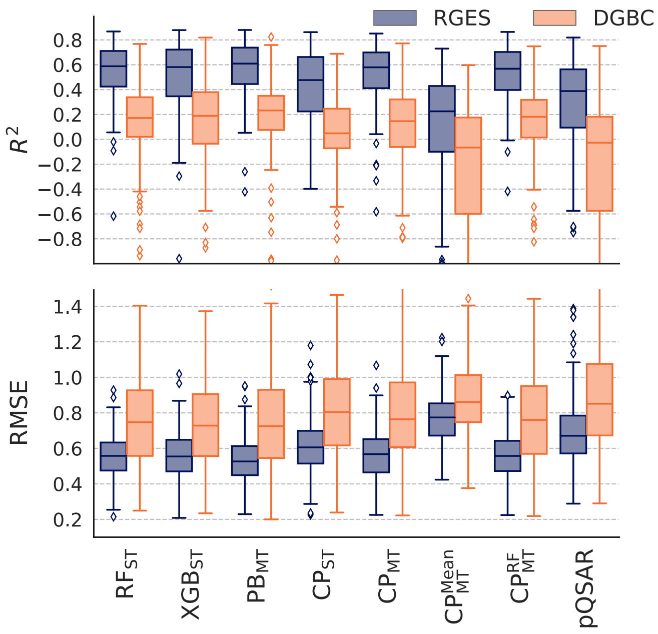

Figure 3: **Comparison of the performance of the different models evaluated both with the random and structure-based splits.** Distributions of R^2 and RMSE values between predictions and experimental values of the data in the test set for each target kinase in the kinase200 dataset split using either random global equilibrated selection (RGES, blue) and the dissimilarity-driven global balanced cluster (DGBC, orange) splits. Predictions were made using single-task random forest models ($\mathrm{RF_{ST}}$), xgboost ($\mathrm{XGB_{ST}}$) and chemprop ($\mathrm{CP_{ST}}$) models, multi-task pyboost (PB), and chemprop multi-task model without imputation ($\mathrm{CP_{MT}}$), with mean imputation ($\mathrm{CP^{Mean}_{MT}}$) and with random forest imputation ($\mathrm{CP^{RF}_{MT}}$), and profile QSAR (pQSAR).

3.2.1 Importance of data splitting

For all models, the performance is significantly better on the random-based split than on the structure-base splits. Most models reach median $R^2 > 0.6$ and RMSE< 0.6, often used thresholds to consider a model to be predictive, based on an RGES split. However, on average the median R^2 is 0.3 lower and the median RMSE is 0.2 higher for the DGBC split than the random split showing that the models do not perform as well on this data split. These results are in line with expectations and previously published results[21,39] and show the importance to assess model performance with a realistic split.

3.2.2 Hyperparameter optimisation improves performance

We optimised the hyperparameters of the tree- and chemprop-based models. The hyperparameters of the tree-based models were optimised separately for each model. To limit computation time, we only optimised the $\mathrm{CP_{MT}}$ model's hyperparameters on the DGBC split and then applied the optimised parameters to all chemprop models. We show the performance of the optimised models in Figure 3 and Table 2, and the performances of the default models are summarised in Table S2.

On the RGES split, the hyperparameter optimisation significantly increases the performance of all models, except the $\mathrm{RF_{ST}}$ models for which the performance does not change. The median R^2 and RMSE were improved by 0.12 and 0.07 on average, respectively, and the largest improvements are seen for the $\mathrm{PB_{MT}}$) model (ΔR^2 =0.30 and ΔRMSE $=$ 0.19). In the case of the DGBC split, the effect of the hyperparameter optimisation is much smaller with the median R^2 and RMSE only improving by 0.07 and 0.03 on average, respectively. The largest improvements are also seen for $\mathrm{PB_{MT}}$ model (ΔR^2=0.16 and ΔRMSE=0.07) and the second largest for the $\mathrm{XGB_{ST}}$ models, indicating that hyperparameter optimisation is necessary for gradient boosting models. It is surprising that the effect is consistently larger in the RGES than the DGBC split for the DMPNN-based models as the hyperparameter optimisation was done with a small subset with the balanced cluster split. Nevertheless, all models were improved by hyperparameter optimisation and future studies could also use algorithms, like FABOLAS,[40] that can handle hyperparameter optimisation on very large datasets to yield even greater gain in performance for the multi-task DMPNN models. The subsequent analyses in this paper were done with the optimised models.

Table 2: **Performance of kinase activity prediction models.** Median, mean and standard deviations of R^2 and RMSE metrics per kinase for single-task random forest ($\mathrm{RF_{ST}}$), xgboost ($\mathrm{XGB_{ST}}$), and chemprop single-task ($\mathrm{CP_{ST}}$) models, multi-task pyboost model ($\mathrm{PB_{MT}}$), chemprop multi-task models without data imputation ($\mathrm{CP_{MT}}$), and with mean ($\mathrm{CP_{MT}^{Mean}}$) and RF imputation ($\mathrm{CP_{MT}^{RF}}$), and the profile QSAR model without data leakage (pQSAR).

	R^2				RMSE			
	Median		Mean (std)		Median		Mean (std)	
	RGES	DGBC	RGES	DGBC	RGES	DGBC	RGES	DGBC
$\mathrm{RF_{ST}}$	0.59	0.17	0.54 (0.22)	0.15 (0.32)	0.56	0.75	0.55 (0.13)	0.77 (0.30)
$\mathrm{XGB_{ST}}$	0.58	0.19	0.51 (0.27)	0.12 (0.64)	0.55	0.73	0.56 (0.13)	0.76 (0.30)
$\mathrm{PB_{MT}}$	**0.61**	**0.23**	**0.57** (0.21)	**0.20** (0.28)	**0.53**	**0.72**	**0.53** (0.13)	**0.75** (0.29)
$\mathrm{CP_{ST}}$	0.48	0.05	0.42 (0.30)	0.03 (0.40)	0.61	0.80	0.61 (0.16)	0.82 (0.25)
$\mathrm{CP_{MT}}$	0.58	0.15	0.52 (0.24)	0.08 (0.40)	0.57	0.76	0.56 (0.14)	0.78 (0.25)
$\mathrm{CP_{MT}^{Mean}}$	0.23	-0.07	0.02 (0.68)	-0.37 (0.99)	0.77	0.86	0.77 (0.13)	0.89 (0.23)
$\mathrm{CP_{MT}^{RF}}$	0.57	0.18	0.54 (0.22)	0.14 (0.33)	0.56	0.76	0.56 (0.13)	0.78 (0.1)
pQSAR	0.39	-0.03	0.26 (0.49)	-0.46 (2.14)	0.67	0.85	0.69 (0.21)	0.94 (0.50)

3.2.3 Multi-task models outperform a collection of singe-task models

To evaluate the effect utilising correlations between kinases in sparse data we compared pairwise the performance of single- and multi-task *(i)* gradient boosting models ($\mathrm{XGB_{ST}}$s vs. $\mathrm{PB_{MT}}$) and *(ii)* chemprop models ($\mathrm{CP_{ST}}$s vs. $\mathrm{CP_{MT}}$). For both cases and both splits, the multi-task models are superior to the set of single-task models with increased average R^2-scores and decreased RMSEs (Table 3).

Table 3: **Effect of multi-task modelling.** Difference between the mean R^2 and RMSE per kinase of single- and multi-task models.

	$\mathrm{XGB_{ST}/PB_{MT}}$		$\mathrm{CP_{ST}/CP_{MT}}$	
	RGES	DGBC	RGES	DGBC
$\Delta\langle R^2\rangle$	0.06	0.08	0.11	0.05
$\Delta\langle\mathrm{RMSE}\rangle$	-0.03	-0.01	-0.05	-0.04

The effects of multi-task models are larger for the deep learning model compared to the gradient-boosting one. The CP models are based on learned representations, therefore on small, single-task datasets, the model is most likely not able to extract generalisable embeddings. The multi-task model uses a much larger dataset from which it is able to extract more generalisable embeddings, leading to a significant improvement. On the R^2-score, on which we see the larger gains, the improvements are larger for RGES and DGBC splits in the case of deep learning and gradient boosting models, respectively. This indicates that the exploitation of inter-target correlation can useful when predicting the activities of compounds dissimilar to the ones in the training set. Furthermore, in the case of the deep learning models, there is a speed-up of a factor ~30 in computation time between running a single 198 multi-task model and running 198 separate single-task models.

Similarly, in a recent study, Moriwaki et al.[41] using an in-house dataset with random splits for binary kinase activity predictions showed promising results for multi-task graph neural networks that outperform single-task models. Another option to exploit the inter-target correlation is proteochemometric modelling.[42] However, both our single- and multi-task models seem to outperform proteochemometric models used by Born et al. (RMSEs above 0.75 in a random split-based evaluation)[26] in their large-scale kinase modelling.

3.2.4 Tree-based machine learning outperforms deep learning

Both in the case of single-task models ($\mathrm{RF_{ST}}$, $\mathrm{XGB_{ST}}$ vs. $\mathrm{CP_{ST}}$) and multi-task models ($\mathrm{PB_{ST}}$ vs. $\mathrm{CP_{MT}}$) , we see that the classical tree-based machine learning methods outperform the

deep learning model. Even though in general deep learning models have been shown to outperform classic machine learning approaches in activity prediction,[43] these results are in line with in-house results and other publications[23,44,45] that show that in some cases classic ML methods perform as well as deep learning models.

The superior performance of tree-based methods over deep learning models has been well described on small- to medium-sized tabular datasets - several reasons for this are: NNs are biased towards overly smooth solutions, uninformative features are more problematic for NNs, NNs are typically not rotationally invariant - as discussed in Ref. 46. Moreover, the deep learning approach is based on learned representations, therefore on small, single-task datasets, the model is most likely not able to extract generalisable embeddings. This would explain why the difference between the single-task models is larger than between the multi-task ones. Furthermore, the hyperparameters of each tree-based model were optimised separately, whereas, due to computation time, deep learning models use parameters optimised once on a subset of the multi-task model so they might not be optimal for each separate model.

3.2.5 Performance is not correlated with data density

We have evaluated whether there is a correlation between density and model performance and whether there is a difference in performance when using datasets at different density levels. In Figure 4 we illustrate the performance of the $\mathrm{CP}_{\mathrm{MT}}$ model for each kinase is poorly correlated with the data density points for that kinase, assessed by the R^2 and the RMSE.

To evaluate how the multi-task model performance is affected by adding activity data on targets that have lower data density, the $\mathrm{CP}_{\mathrm{MT}}$ was run on two datasets, kinase1000 and kinase200, which have different densities. The performance difference for targets that are both present in the kinase200 and kinase1000 datasets is shown by the distributions of $\Delta R^2 = R^2_{\mathrm{kinase1000}} - R^2_{\mathrm{kinase200}}$ and $\Delta\mathrm{RMSE} = \mathrm{RMSE}_{\mathrm{kinase1000}} - \mathrm{RMSE}_{\mathrm{kinase200}}$ in Figure 5. For the RGES split it is clear that

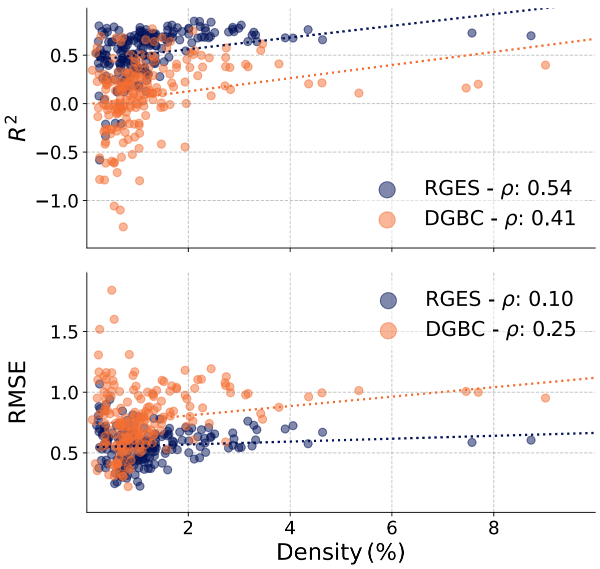

Figure 4: $\mathrm{CP}_{\mathrm{MT}}$**'s performance per kinase as a function of kinase's data density.** The coefficient of determination (R^2) and root-mean-squared error (RMSE) between predictions and experimental values for the test set of each target kinase as a function of the data density of that target kinase. Results for random global equilibrated selection (RGES) split in blue and the dissimilarity-driven global balanced cluster (DGBC) split in orange.

there is no difference in performance between the two datasets, so adding activity data from targets that have fewer data points does not improve the performance of the models on the other targets. For the DGBC split, there are larger differences in performance between sets, but there is no systematic improvement with the addition of the sparser kinases to the dataset. Overall, adding more kinases with fewer data points leads to a sparser data matrix, and it does not improve model performance.

3.2.6 Data imputation does not improve chemprop performance

As the dataset is very sparse, making it difficult to exploit inter-kinase correlation, we investigated if data imputation could improve the performance of the multi-task models. Using chemprop, multi-tasking methods *with* imputation, $\mathrm{CP}^{\mathrm{Mean}}_{\mathrm{MT}}$ and $\mathrm{CP}^{\mathrm{RF}}_{\mathrm{MT}}$, does not show any improvement over multi-tasking *without* imputation $\mathrm{CP}_{\mathrm{MT}}$ (Figure 3

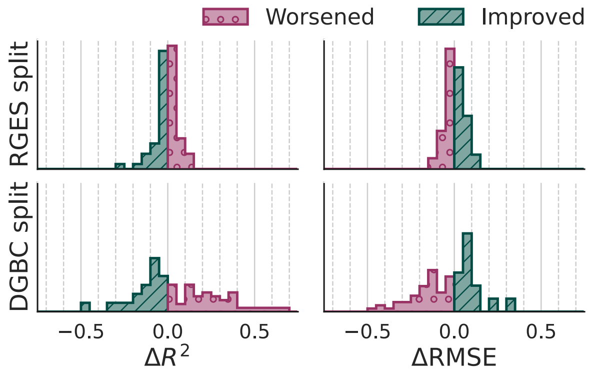

Figure 5: **Difference of $\mathrm{CP_{MT}}$'s performance per kinase between the smaller and larger dataset.** Distributions of $R^2 = R^2_{\mathrm{kinase1000}} - R^2_{\mathrm{kinase200}}$ (top) and for $\mathrm{RMSE} = \mathrm{RMSE}_{\mathrm{kinase1000}} - \mathrm{RMSE}_{\mathrm{kinase200}}$ (bottom) for kinase present in both datasets. In green and purple, kinases for which model performance increases or decreases with the addition of sparse kinases to the dataset.

and Table 2). $\mathrm{CP^{RF}_{MT}}$ has very similar performance to $\mathrm{CP_{MT}}$ and $\mathrm{CP^{Mean}_{MT}}$ underperforms significantly compared any other model, single- or multi-task.

3.2.7 pQSAR without data leakage underperforms

In Martin, et al.[27] the test set is included when training the PLS model which leads to data leakage between kinases. To investigate the impact of this, we trained the PLS both with and without the test set. In Figure 6 and Table S3 we show that when the test set is not included in the training of the PLS, the performance measures turn out worse than when the test set is included. The pQSAR without the test set performs worse than the single-task $\mathrm{RF_{ST}}$s, and when the test set is included in training it outperforms the RFs. The test set should be independent so we exclude the test set from the training of the PLS models. In this scenario, the pQSAR model underperforms compared to every other model except $\mathrm{CP^{Mean}_{MT}}$. Alchemite, a commercial deep learning-based method using imputation adopts an expectation-maximisation algorithm and has shown promising results on the cluster-based pQSAR dataset as well, but as mentioned before the splits from pQSAR suffer from data-leakage.[20]

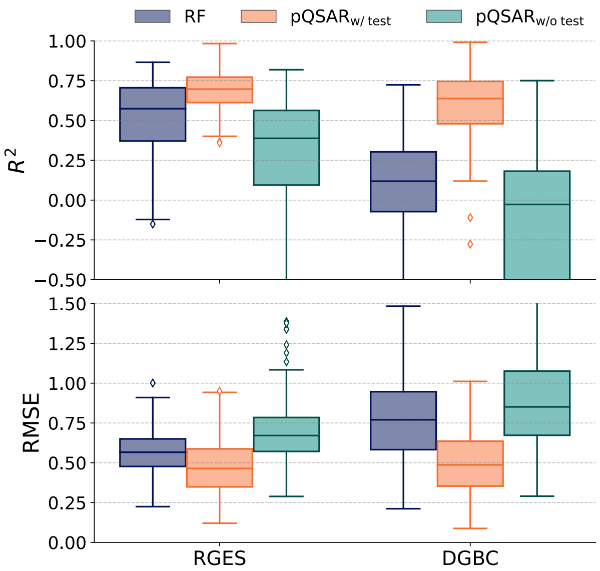

Figure 6: **Performance of pQSAR performance with and without data leakage.** Distributions of R2 and RMSE values between predictions and experimental values of the data in the test set for each target kinase in the kinase200 dataset. Predictions were made using single task random forest models ($\mathrm{RF_{ST}}$), pQSAR with PLS trained on training and test set (pQSAR$_{\text{w/ test}}$) and profile QSAR with PLS trained on the training set only (pQSAR$_{\text{w/o test}}$). Results are shown both for the random global equilibrated split (RGES) or dissimilarity-driven global balanced cluster (DGBC) split.

4 Conclusion

In this study, we have investigated the large-scale modelling of protein kinase activity with various machine-learning approaches. For this, we created two large protein kinase datasets from the curated Papyrus database,[31] comprising 198 kinases and 83K molecules (kinase200) and 66 kinases and 71K molecules (kinase1000), respectively. Other recently applied kinase datasets contain either a more limited number of targets[30] or molecules,[27,29] except for the dataset based on all human kinases used by Born et al.[26] For each of our datasets, two balanced multi-task splits without data leakage between targets were created, a random- (RGES) and a dissimilarity-driven

cluster-based (DGBC) split, to evaluate rigorously the performance of the models within exploitation and exploration schemes. Other publications of large-scale kinase modelling have been using either only random-based splits[26] or have had data leakage between targets.[27]

We then compared the performance of seven models to predict protein kinase activity. These included sets of single-task random forest, xgboost and chemprop models, multi-task pyboost model, chemprop models – without and with data imputation – and our implementation of pQSAR 2.0, Both in the cases of single and multi-task models, we see that the deep learning-based models are outperformed by classic machine learning methods on both splits. The performance of gradient boosting and D-MPNN models on both splits can be improved by creating multi-task models, indicating that exploitation of inter-target correlation can be useful when predicting activities of compounds both similar and dissimilar to the ones in the training set. The multi-task model's performance could not be improved by data imputation. Moreover, using pQSAR without data leakage between targets also does not lead to higher predictive power.

As expected, we see that every model performs significantly worse with the dissimilarity-driven split than with the random split. Most of the models show some predictive power with the random split but struggle with the cluster-based split. The dissimilarity-driven cluster-based split is a more conservative estimation of performance and thus it is a more realistic assessment of the performance of machine learning models in real drug discovery projects. As the poor performance on the cluster-based split of the different models shows (for the $\mathrm{PB}_{\mathrm{MT}}$, the overall best-performing model, only 19% of kinase have an R^2 above 0.4), further developments are needed to efficiently model activity with large-scale sparse datasets. The inclusion of additional data in the form of protein information may improve this performance.

Glossary

density	(or data density) percentage of a data matrix fil
CP	chemprop
DGBC	dissimilarity-driven global balance clustering
DMPNN	directed message passing neural network
MT	multi-task
PB	pyboost
R^2	coefficient of determination
RF	random forest
RGES	random global equilibrated selection
RMSE	root-mean-squared error
ST	single-task
XGB	xgboost

Data and Software Availability

The code and the datasets are provided at to the community at https://github.com/CDDLeiden/kinase-modelling as a benchmark set for developing large-scale multi-task models for kinases.

5 Supporting Information Available

Available free of charge. S1 - Extra analysis of the kinase200 split, S2 - analysis of the kinase1000 splits, S3 - models' performance with default parameters on kinase200 set, S4 - models' performance with default parameters on kinase1000 set, S5 - pQSAR implementation validation.

Author contributions

SL: Validation, Formal analysis, Writing – Original Draft, Writing - Review & Editing, Visualization, Supervision
EM: Methodology, Software, Formal analysis, Investigation, Writing – Original Draft, Visualization
GAT: Methodology, Software
JH: Software, Infrastructure
PFWS: Writing - Review & Editing, Supervision, Funding acquisition
GJPvW: Resources, Writing - Review & Editing, Supervision, Funding acquisition

EBL: Conceptualization, Methodology, Software, Writing - Review & Editing, Supervision

Acknowledgement SL and EM thank Olivier Béquignon for his help with Papyrus and many fruitful discussions, and Roelof van der Kleij for his help using the university IT infrastructure. SL also thanks her Bachelor students, Niels Tenseling and Kian Noorman van der Dussen. This research received funding from the Dutch Research Council (NWO) in the framework of the Science PPP Fund for the top sectors and acknowledges the Dutch Research Council (NWO ENPPS.LIFT.019.010).

Supporting Information Available

Available free of charge: S1 - Extra analysis of the kinase200 split, S2 - analysis of the kinase1000 splits, S3 - models' performance with default parameters on kinase200 set, S4 - models' performance with default parameters on kinase1000 set, S5 - pQSAR implementation validation.

References

(1) Bossemeyer, D. Protein kinases — structure and function. *FEBS Letters* **1995**, *369*, 57–61, DOI: 10.1016/0014-5793(95)00580-3.

(2) Manning, G.; Whyte, D. B.; Martinez, R.; Hunter, T.; Sudarsanam, S. The Protein Kinase Complement of the Human Genome. *Science* **2002**, *298*, 1912–1934, DOI: 10.1126/science.1075762.

(3) Cock, J. M.; Vanoosthuyse, V.; Gaude, T. Receptor kinase signalling in plants and animals: distinct molecular systems with mechanistic similarities. *Curr. Opin. Cell Biol.* **2002**, *14*, 230–236, DOI: 10.1016/S0955-0674(02)00305-8.

(4) Cohen, P. The regulation of protein function by multisite phosphorylation – a 25 year update. *Trends Biochem. Sci.* **2000**, *25*, 596–601, DOI: 10.1016/S0968-0004(00)01712-6.

(5) Levitzki, A. Protein Kinase Inhibitors as a Therapeutic Modality. *Acc. Chem. Res.* **2003**, *36*, 462–469, DOI: 10.1021/ar0201207.

(6) Cohen, P.; Cross, D.; Jänne, P. A. Kinase drug discovery 20 years after imatinib: progress and future directions. *Nat. Rev. Drug Discov.* **2021**, *20*, 551–569, DOI: 10.1038/s41573-021-00195-4.

(7) Lu, X.; Smaill, J. B.; Ding, K. New Promise and Opportunities for Allosteric Kinase Inhibitors. *Angew. Chem. Int* **2020**, *59*, 13764–13776, DOI: 10.1002/anie.201914525.

(8) DiMasi, J. A.; Grabowski, H. G.; Hansen, R. W. Innovation in the pharmaceutical industry: New estimates of R&D costs. *J. Health Econ.* **2016**, *47*, 20–33, DOI: 10.1016/j.jhealeco.2016.01.012.

(9) Muratov, E. N.; Bajorath, J.; Sheridan, R. P.; Tetko, I. V.; Filimonov, D.; Poroikov, V.; Oprea, T. I.; Baskin, I. I.; Varnek, A.; Roitberg, A.; Isayev, O.; Curtalolo, S.; Fourches, D.; Cohen, Y.; Aspuru-Guzik, A.; Winkler, D. A.; Agrafiotis, D.; Cherkasov, A.; Tropsha, A. QSAR without borders. *Chem. Soc. Rev.* **2020**, *49*, 3525–3564, DOI: 10.1039/D0CS00098A.

(10) Wilhelm, S. M.; Adnane, L.; Newell, P.; Villanueva, A.; Llovet, J. M.; Lynch, M. Preclinical overview of sorafenib, a multikinase inhibitor that targets both Raf and VEGF and PDGF receptor tyrosine kinase signaling. *Mol. Cancer Ther.* **2008**, *7*, 3129–3140, DOI: 10.1158/1535-7163.MCT-08-0013.

(11) Kinnings, S. L.; Jackson, R. M. Binding Site Similarity Analysis for the Functional Classification of the Protein Kinase Family. *J. Chem. Info. Model.* **2009**, *49*, 318–329, DOI: 10.1021/ci800289y.

(12) Kornev, A. P.; Taylor, S. S. Defining the conserved internal architecture of a protein kinase. *Biochim. Biophys. Acta - Proteins Proteom.* **2010**, *1804*, 440–444, DOI: 10.1016/j.bbapap.2009.10.017.

(13) Tang, J.; Szwajda, A.; Shakyawar, S.; Xu, T.; Hintsanen, P.; Wennerberg, K.; Aittokallio, T. Making Sense of Large-Scale Kinase Inhibitor Bioactivity Data Sets: A Comparative and Integrative Analysis. *J. Chem. Info. Model.* **2014**, *54*, 735–743, DOI: 10.1021/ci400709d.

(14) Backman, T. W. H.; Girke, T. bioassayR: Cross-Target Analysis of Small Molecule Bioactivity. *J. Chem. Info. Model.* **2016**, *56*, 1237–1242, DOI: 10.1021/acs.jcim.6b00109.

(15) de la Vega de León, A.; Chen, B.; Gillet, V. J. Effect of missing data on multitask prediction methods. *J. Cheminform.* **2018**, *10*, 26, DOI: 10.1186/s13321-018-0281-z.

(16) Zhao, Z.; Qin, J.; Gou, Z.; Zhang, Y.; Yang, Y. Multi-task learning models for predicting active compounds. *J. Biomed. Inform.* **2020**, *108*, 103484, DOI: 10.1016/j.jbi.2020.103484.

(17) Jadhav, A.; Pramod, D.; Ramanathan, K. Comparison of Performance of Data Imputation Methods for Numeric Dataset. *Appl. Artif. Intell.* **2019**, *33*, 913–933, DOI: 10.1080/08839514.2019.1637138.

(18) Irwin, B. W. J.; Mahmoud, S.; Whitehead, T. M.; Conduit, G. J.; Segall, M. D. Imputation versus prediction: applications in machine learning for drug discovery. *Future Drug Discovery* **2020**, *2*, FDD38, DOI: 10.4155/fdd-2020-0008.

(19) Walter, M.; Allen, L. N.; de la Vega de León, A.; Webb, S. J.; Gillet, V. J. Analysis of the benefits of imputation models over traditional QSAR models for toxicity prediction. *J. Cheminform.* **2022**, *14*, 32, DOI: 10.1186/s13321-022-00611-w.

(20) Whitehead, T. M.; Irwin, B. W. J.; Hunt, P.; Segall, M. D.; Conduit, G. J. Imputation of Assay Bioactivity Data Using Deep Learning. *J. Chem. Info. Model.* **2019**, *59*, 1197–1204, DOI: 10.1021/acs.jcim.8b00768.

(21) Tricarico, G. A.; Hofmans, J.; Lenselink, E. B.; López-Ramos, M.; Dréanic, M.-P.; Stouten, P. F. W. Construction of balanced, chemically dissimilar training, validation and test sets for machine learning on molecular datasets. **2022**, DOI: 10.26434/chemrxiv-2022-m8l33-v2.

(22) Sheridan, R. P. Time-Split Cross-Validation as a Method for Estimating the Goodness of Prospective Prediction. *J. Chem. Info. Model.* **2013**, *53*, 783–790, DOI: 10.1021/ci400084k.

(23) Cichońska, A.; Ravikumar, B.; Allaway, R. J.; Wan, F.; Park, S.; Isayev, O.; Li, S.; Mason, M.; Lamb, A.; Tanoli, Z.; Jeon, M.; Kim, S.; Popova, M.; Capuzzi, S.; Zeng, J.; Dang, K.; Koytiger, G.; Kang, J.; Wells, C. I.; Willson, T. M.; Oprea, T. I.; Schlessinger, A.; Drewry, D. H.; Stolovitzky, G.; Wennerberg, K.; Guinney, J.; Aittokallio, T. Crowdsourced mapping of unexplored target space of kinase inhibitors. *Nat. Commun.* **2021**, *12*, 3307, DOI: 10.1038/s41467-021-23165-1.

(24) Niijima, S.; Shiraishi, A.; Okuno, Y. Dissecting Kinase Profiling Data to Predict Activity and Understand Cross-Reactivity of Kinase Inhibitors. *J. Chem. Info. Model.* **2012**, *52*, 901–912, DOI: 10.1021/ci200607f.

(25) Christmann-Franck, S.; van Westen, G. J. P.; Papadatos, G.; Beltran Escudie, F.; Roberts, A.; Overington, J. P.; Domine, D. Unprecedently Large-Scale Kinase Inhibitor Set Enabling the Accurate Prediction of Compound–Kinase Activities: A Way toward Selective Promiscuity by Design? *J. Chem. Info. Model.* **2016**, *56*, 1654–1675, DOI: 10.1021/acs.jcim.6b00122.

(26) Born, J.; Huynh, T.; Stroobants, A.; Cornell, W. D.; Manica, M. Active Site Sequence Representations of Human Kinases Outperform Full Sequence Representations for Affinity Prediction and Inhibitor Generation: 3D Effects in a 1D Model. *J. Chem. Info. Model.* **2022**, *62*, 240–257, DOI: 10.1021/acs.jcim.1c00889.

(27) Martin, E. J.; Polyakov, V. R.; Tian, L.; Perez, R. C. Profile-QSAR 2.0: Kinase Virtual Screening Accuracy Comparable to Four-Concentration IC50s for Realistically Novel Compounds. *J. Chem. Info. Model.* **2017**, *57*, 2077–2088, DOI: 10.1021/acs.jcim.7b00166.

(28) Metz, K. S.; Deoudes, E. M.; Berginski, M. E.; Jimenez-Ruiz, I.; Aksoy, B. A.; Hammerbacher, J.; Gomez, S. M.; Phanstiel, D. H. Coral: Clear and Customizable Visualization of Human Kinome Data. *Cell Syst.* **2018**, *7*, 347–350.e1, DOI: 10.1016/j.cels.2018.07.001.

(29) Elkins, J. M.; Fedele, V.; Szklarz, M.; Abdul Azeez, K. R.; Salah, E.; Mikolajczyk, J.; Romanov, S.; Sepetov, N.; Huang, X.-P.; Roth, B. L.; Al Haj Zen, A.; Fourches, D.; Muratov, E.; Tropsha, A.; Morris, J.; Teicher, B. A.; Kunkel, M.; Polley, E.; Lackey, K. E.; Atkinson, F. L.; Overington, J. P.; Bamborough, P.; Müller, S.; Price, D. J.; Willson, T. M.; Drewry, D. H.; Knapp, S.; Zuercher, W. J. Comprehensive characterization of the Published Kinase Inhibitor Set. *Nat. Biotechnol.* **2016**, *34*, 95–103, DOI: 10.1038/nbt.3374.

(30) Sharma, R.; Schürer, S. C.; Muskal, S. M. *High quality, small molecule-activity datasets for kinase research*; 2016; DOI: 10.12688/f1000research.8950.3.

(31) Béquignon, O. J. M.; Bongers, B. J.; Jespers, W.; IJzerman, A. P.; van der Water, B.; van Westen, G. J. P. Papyrus: a large-scale curated dataset aimed at bioactivity predictions. *J. Cheminform.* **2023**, *15*, 3, DOI: 10.1186/s13321-022-00672-x.

(32) Landrum, G. Thresholds for "random" in fingerprints the RDKit supports. 2021; https://github.com/greglandrum/rdkit_blog/blob/master/notebooks/Fingerprint%20Thresholds.ipynb.

(33) Landrum, G.; Tosco, P.; Kelley, B.; Ric,; sriniker,; gedeck,; Vianello, R.; NadineSchneider,; Kawashima, E.; Cosgrove, D.; Dalke, A.; N, D.; Jones, G.; Cole, B.; Swain, M.; Turk, S.; AlexanderSavelyev,; Vaucher, A.; Wójcikowski, M.; Take, I.; Probst, D.; Ujihara, K.; Scalfani, V. F.; guillaume godin,; Pahl, A.; Berenger, F.; JLVarjo,; strets123,; JP,; DoliathGavid, rdkit/rdkit: 2022_03_5 (Q1 2022) Release. 2022; https://doi.org/10.5281/zenodo.6961488.

(34) Yang, K.; Swanson, K.; Jin, W.; Coley, C.; Eiden, P.; Gao, H.; Guzman-Perez, A.; Hopper, T.; Kelley, B.; Mathea, M.; Palmer, A.; Settels, V.; Jaakkola, T.; Jensen, K.; Barzilay, R. Analyzing Learned Molecular Representations for Property Prediction. *J. Chem. Info. Model.* **2019**, *59*, 3370–3388, DOI: 10.1021/acs.jcim.9b00237.

(35) Pedregosa, F.; Varoquaux, G.; Gramfort, A.; Michel, V.; Thirion, B.; Grisel, O.; Blondel, M.; Prettenhofer, P.; Weiss, R.; Dubourg, V.; Vanderplas, J.; Passos, A.; Cournapeau, D.; Brucher, M.; Perrot, M.; Duchesnay, E. Scikit-learn: Machine Learning in Python. *J. Mach. Learn. Res.* **2011**, *12*, 2825–2830.

(36) Chen, T.; Guestrin, C. XGBoost: A Scalable Tree Boosting System. Proceedings of the 22nd ACM SIGKDD International Conference on Knowledge Discovery and Data Mining. 2016; p 785–794, DOI: 10.1145/2939672.2939785.

(37) Iosipoi, L.; Vakhrushev, A. SketchBoost: Fast Gradient Boosted Decision Tree for Multioutput Problems. Advances in Neural Information Processing Systems. 2022; pp 25422–25435.

(38) Akiba, T.; Sano, S.; Yanase, T.; Ohta, T.; Koyama, M. Optuna: A Next-generation Hyperparameter Optimization Framework. Proceedings of the 25rd ACM SIGKDD International Conference on Knowledge Discovery and Data Mining. 2019; DOI: 10.1145/3292500.3330701.

(39) Masand, V. H.; Mahajan, D. T.; Nazeruddin, G. M.; Hadda, T. B.; Rastija, V.;

Alfeefy, A. M. Effect of information leakage and method of splitting (rational and random) on external predictive ability and behavior of different statistical parameters of QSAR model. *Med. Chem. Res.* **2015**, *24*, 1241–1264, DOI: 10.1007/s00044-014-1193-8.

(40) Klein, A.; Falkner, S.; Bartels, S.; Hennig, P.; Hutter, F. Fast Bayesian hyperparameter optimization on large datasets. *Electron. J. Stat.* **2017**, *11*, 4945–4968, DOI: 10.1214/17-EJS1335SI.

(41) Moriwaki, H.; Saito, S.; Matsumoto, T.; Serizawa, T.; Kunimoto, R. Global Analysis of Deep Learning Prediction Using Large-Scale In-House Kinome-Wide Profiling Data. *ACS Omega* **2022**, *7*, 18374–18381, DOI: 10.1021/acsomega.2c00664.

(42) Bongers, B. J.; IJzerman, A. P.; van Westen, G. J. P. Proteochemometrics – recent developments in bioactivity and selectivity modeling. *Drug Discov. Today Technol.* **2019**, *32-33*, 89–98, DOI: 10.1016/j.ddtec.2020.08.003.

(43) Lenselink, E. B.; ten Dijke, N.; Bongers, B.; Papadatos, G.; van Vlijmen, H. W. T.; Kowalczyk, W.; IJzerman, A. P.; van Westen, G. J. P. Beyond the hype: deep neural networks outperform established methods using a ChEMBL bioactivity benchmark set. *J. Cheminform.* **2017**, *9*, 45, DOI: 10.1186/s13321-017-0232-0.

(44) Sheridan, R. P.; Wang, W. M.; Liaw, A.; Ma, J.; Gifford, E. M. Extreme Gradient Boosting as a Method for Quantitative Structure–Activity Relationships. *J. Chem. Info. Model.* **2016**, *56*, 2353–2360, DOI: 10.1021/acs.jcim.6b00591.

(45) Siemers, F. M.; Feldmann, C.; Bajorath, J. Minimal data requirements for accurate compound activity prediction using machine learning methods of different complexity. *Cell Reports Physical Science* **2022**, *0*, DOI: 10.1016/j.xcrp.2022.101113.

(46) Grinsztajn, L.; Oyallon, E.; Varoquaux, G. Why do tree-based models still outperform deep learning on tabular data? 2022; http://arxiv.org/abs/2207.08815, arXiv:2207.08815 [cs, stat].

TOC Graphic

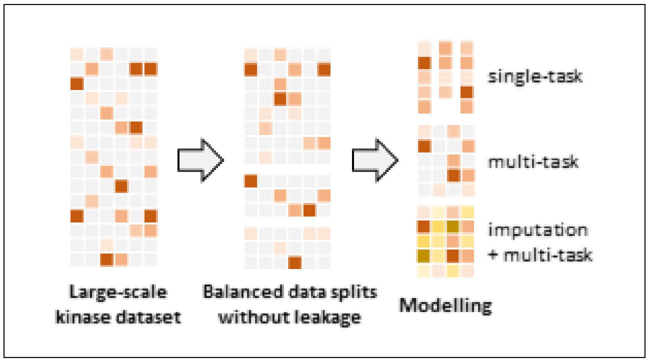
single-task
multi-task
imputation
+ multi-task
Large-scale
kinase dataset
Balanced data splits
without leakage
Modelling
